# 2 Novel deletions of the sterol 27-hydroxylase gene in a Chinese Family with Cerebrotendinous Xanthomatosis

**DOI:** 10.1186/1471-2377-11-130

**Published:** 2011-10-21

**Authors:** Di Tian, Zai-qiang Zhang

**Affiliations:** 1 Department of Neurology, Beijing Tiantan Hospital, Capital Medical University, 6 Tiantan Xili, Chongwen District, Beijing 100050, China

**Keywords:** Cerebrotendinous Xanthomatosis, Sterol 27-hydroxylase Gene, CYP27A1

## Abstract

**Background:**

Cerebrotendinous xanthomatosis (CTX) is a rare lipid-storage disease. We investigated the clinic manifestation, histopathology and sterol 27-hydroxylase gene (CYP27A1) in a Chinese family with Cerebrotendinous Xanthomatosis (CTX).

**Case Presentation:**

A 36-year-old female with typical CTX clinical manifestation had Spindle-shaped lipid crystal clefts in xanthomas and "onion-like demyelination" in sural nerve. The patient was compound heterozygote carrying two deletions in exon 1 (c.73delG) and exon 2 (c.369_375delGTACCCA). The family memebers were carriers.

**Conclusions:**

A Chinese family with Cerebrotendinous Xanthomatosis had typical clinical manifestation. CYP27A1 mutations were found in the proband and all other family members.

## Background

Cerebrotendinous Xanthomatosis (CTX) is a rare autosomal recessive sterol storage disease caused by a mutated sterol 27-hydroxylase gene (CYP27A1) [1]. The CYP27A1 gene is located on chromosome 2q33-qter and consists of 9 exons. Sterol 27-hydroxylase is a mitochondrial cytochrome P 450 enzyme that catalyzes the initial steps in the oxidation of side chain of sterol intermediates in the pathway leading to the formation of bile acids in the liver [2]. As the normal pathway is impaired, cholesterol and cholestanol accumulate at central nervous system, tendon, lenses, lung and bones [3]. Many cases were reported all around the world, and more than 50 mutations of CYP27A1 were reported in the literature [4-8]. Here we describe clinical findings, neuro-imaging, pathology and two novel mutations of the CYP27A1 gene in a Chinese family.

## Case presentation

A 36 year-old Chinese female had a disturbance of gait, recurrent diarrhea and bilateral cataracts when she was 7 years old. Because of the poor eyesight she dropped out of school. When she was 13 years old, she had cognitive impairments, and developed digestive symptom of diarrhea initially and constipation later. At the age of 27 years, she spoke unclearly, had weakness of bilateral lower limbs and found swelling of Achilles tendon. She could still walk unassisted. As she was 32 years old, her head swayed left-to-right involuntarily, which was prominent while sleeping. When 34 years old, dry cough at night and dysphagia appeared. She could not walk at the age of 35 years and her right knee joint luxated three times when she tried to stand. There was no known consanguinity in previous generations.

**Figure 1:**
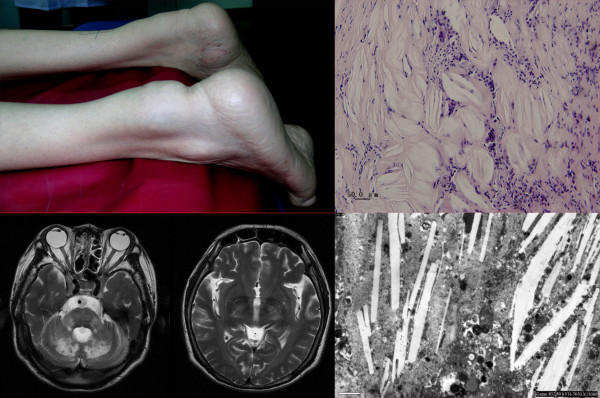
**Achilles tendon xanthomas (A) and hyperintensive signals on both sides of internal capsule, pons and dentate nucleus at MRI (B) as typical CTX findings**. The lesions of spindle-shaped lipid crystal clefts in H-E staining (C) and rod-like cholesterol deposit in the electron microscope (D) indicated the diagnosis of CTX.

Tendon xanthomas were observed in both elbows, right dorsal foot, and both Achilles tendons (Figure 1A). The teeth were irregular and destroyed. She was alert, but unable to repeat or comprehend complex phrases. She had difficulty with recall at five minutes and unable to name the months backwards. Speech was dysarthic. Plantar responses were both extensor. She had severe ataxia, dysdiadochokinesia and dysmetria. The strength of distal limbs was 4/5. The vibratory sensation was slightly diminished in the lower limbs.

Cholesterol was 3.67 mmol/L (3.2-5.17 mmol/L), HDL 1.09 mmol/L(1.0-1.8 mmol/L), and LDL 2.03 mmol/L (1.5-3.1 mmol/L). The chest X-ray showed left lung hyperdense with clear round boundary. T2-weighted MRI of brain showed hyperintensive signals on both sides of the internal capsule, cerebral peduncle, pons and cerebellar hemisphere (Figure 1B). Biopsy of xanthomas and sural nerve was done to confirmed the diagnosis, H-E staining (Figure 1C) and electron microscope (Figure 1D) showed the typical lesion of spindle-shaped lipid crystal clefts.

Treatment with chenodeoxycholic acid, atorvastatin and aspirin was initiated. Two weeks later, the patient could stand holding on to the bed, and her cough abated. The dorsi and plantar flexion of ankle improved to grade 5-/5.

### Genetic analysis

Written informed consent was obtained from all the family members. The Ethics committee of our hospital approved the study protocol. Blood samples were collected from the patient and her family. Genomic DNA was isolated from peripheral blood leukocytes according to standard procedures and was used as a template for PCR. The sequence information was obtained from Ensembl Genome Browser. 9 exons of the CYP27A1 were amplified by PCR. The primers were designed with Primer3 at internet. For the sequence of exon 1, we used following oligonucletides:5'CCG ATT TCG AAA GAA TCT CG 3', 5'GCA GCC TTC ACT TTC TGT CC3'. For exon 2 we used:5' CTC TGG AAC AAC AGG CCA TC 3', 5'AGA CCA TCA GGC TCA GAG GA 3'. The PCR products were sequenced directly (Invitrogen, Shanghai, China).

## Discussion

**Figure 2:**
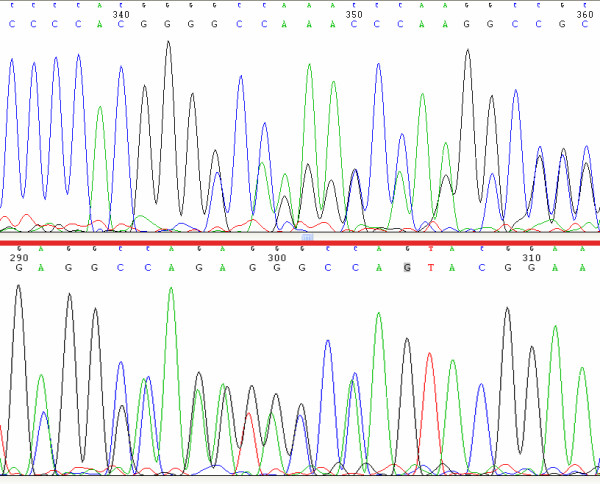
**Exon1(c.73delG) (A) and exon2(c.369-375delGTACCCA) (B) in a Chinese CTX patient**. The patient was a compound heterozygote carrying two mutations in exon 1 (c.73delG) and exon 2 (c.369_375delGTACCCA) (Figure 2). Neither deletion mutation above was reported before. As showed in Figure 3, the I1, III1 and III2 are the carriers of c.369_375delGTACCCA, while I2 and III3 are the carriers of c.73delG.

**Figure 3:**
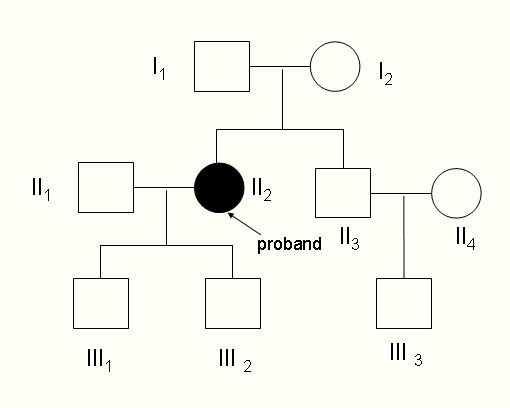
**I1, III1 and III2 are the carrier of c.369_375delGTACCCA**. I2 and III3 are the carrier of c.73delG. II2 is the proband. DNA of II1, II3 and II4 were not available.

The sequence analysis of CYP27A1 gene in our CTX patient revealed two mutations, leading to the formation of truncated proteins. The first mutation is a deletion of one nucleotide in exon 1 (c.73delG) generating a frameshift with a premature termination codon (Figure 2A) and resulting in a truncated protein of 56 amino acids. The second mutation is a deletion of seven nucleotides in exon 2 (c.369_375delGTACCCA) generating a frameshift with a premature termination codon (Figure 2B) and resulting in a truncated protein of 196 amino acids. To our knowledge these are not reported in CYP27A1 gene before. We can ensure the proband received exon 2 mutation from her father and exon 1 mutation from her mother (Figure 3). It is difficult to diagnose the disease clinically before 20 years old, when xanthomas usually appear. Genetic analyses could detect an asymptomatic patient in the family for earlier therapeutic intervention.

## Conclusions

A Chinese family with Cerebrotendinous Xanthomatosis had typical clinical manifestation. CYP27A1 mutations were found in the proband and all other family members. Genetic analyses should be needed by asymptomatic patients in the family.

## Competing interests

The authors declare that they have no competing interests.

## Authors' contributions

All authors have read and approved the final manuscript. DThad substantial contributions to conception and design, data acquisition and analysis, drafting the manuscript and revising the manuscript. ZQZhad substantial contributions to conception and design, data analysis, critical revision and final approval of the revision.

## Pre-publication history

The pre-publication history for this paper can be accessed here:

http://www.biomedcentral.com/1471-2377/11/130/prepub
